# Enhancing the Gelation Behavior of Transglutaminase-Induced Soy Protein Isolate(SPI) through Ultrasound-Assisted Extraction

**DOI:** 10.3390/foods13050738

**Published:** 2024-02-28

**Authors:** Gaolin Li, Ran Tao, Yufeng Sun, Lili Wang, Yurui Li, Bei Fan, Fengzhong Wang

**Affiliations:** 1 Institute of Food Science and Technology, Chinese Academy of Agricultural Sciences, Beijing 100193, China; 18854803016@163.com (G.L.); ran.tao5@mail.mcgill.ca (R.T.); yufengsuncaas@163.com (Y.S.); wlland2013@163.com (L.W.); yvruili@163.com (Y.L.); 2 Key Laboratory of Agro-Products Quality and Safety Control in Storage and Transport Process, Ministry of Agriculture and Rural Affairs, Beijing 100193, China

**Keywords:** SPI, ultrasonic modification, physicochemical properties, gel properties

## Abstract

Gelation, as an important functional property of soy protein isolate (SPI), can be improved by some green technologies in food manufacturing, including ultrasound, ultrahigh pressure and microwave treatments. This work investigated the effect of an alkaline solubilisation step in SPI extraction combined with sonication on protein properties. The TGase-induced gel of the modified SPI was prepared to explore the effect of ultrasound on gel properties, including structures, strength, water-holding capacity and rheological properties. Additionally, the differences between traditional ultrasound modification of SPI and current modification methods were analyzed. The results showed that the ultrasonication-assisted extraction method could result in a significant increase in extraction rate from 24.68% to 42.25%. Moreover, ultrasound-assisted modification of SPI gels induced with transglutaminase (TGase) exhibited significant improvement in mechanical properties, such as texture, water-holding capacity and rheological properties, In particular, SPI extracted at 400 W ultrasound intensity for 180 s showed the best overall performance in terms of gel properties. Our method efficiently uniformizes gel structure, enhancing mechanical properties compared to conventional ultrasound methods, which reduced energy consumption and costs. These findings provide insights into the production of high-gelation SPI in food manufacturing.

## 1. Introduction

Soy Protein Isolate (SPI), a widely sourced and versatile protein, is known for its high protein concentration, rich amino acid profile, low saturated fat and substitutability. It offers substantial benefits in the food, health, pharmaceutical and cosmetics industries because of its functional qualities, which include gelation, emulsification and foaming [1]. Three-dimensional network structure, one of the significant advantages of the gel properties of SPI, provides an excellent performance in several applications [2]. Its gelation property can be used to enhance the texture, stability and storage properties of products. In the manufacture of vegetarian and alternative meat products, soy isolate protein gels can mimic meat and provide a high-quality source of protein. In addition, soy protein gel is widely applied as pasta additives, beverages and nutraceuticals that aim at providing its versatility in different manufacturing processes [3]. However, due to the limitation of structure, the traditional heat-induced gelation method has deficiencies in increasing gel strength, water retention ability and other factors, which cannot meet the production requirements. To solve this problem, enzymes or ionic reagents, such as TGase or Ca^2+^, are often used to induce SPI gels, which are able to initiate modification reactions under relatively mild conditions to achieve improved protein functionality. TGase catalyzes the conversion of soluble proteins into three-dimensional structure polymers into a dense and comprehensive network, contributing to enhance gel strength and stability [4]. As it is temperature-independent of TGase-induced amide formation, it provides a greater flexibility in gel formation under milder conditions [5]. Meanwhile, maintaining the natural structure and functionality of SPI is particularly important in applications such as substituting animal proteins, enhancing the nutritional value of foods by increasing the protein content, such as in cereal bars, energy bars, or pH-sensitive food formulations.

Ultrasound technology, being a non-isothermal processing method, finds extensive use in the realm of food processing, especially in modifying the physicochemical characteristics of various food components [6,7]. It is extensively used in the soy protein modification process, offering an efficient and controllable means of protein alteration due to harnessing high-frequency sound waves. In the context of soy protein modification, ultrasonic treatment enhances solubility by inducing conformational changes at the molecular level. It also influences the physical and chemical structure of soy proteins, affecting secondary and tertiary structures. According to Hu et al. [3], ultrasonication increases the solubility of SPI dispersions by promoting the partial unfolding of SPI molecules. It increases the amount of free sulfhydryl (SH) and surface hydrophobicity (H_0_), while decreasing particle size. Khatkar et al. [8] discovered that ultrasonic treatment improved the hydration, thermal properties and in vitro protein digestibility of soy protein. Ultrasound can promote the increased exposure of active groups, facilitating chemical reactions or enzymatic modifications. Moreover, ultrasonic treatment accelerates reaction rates through the creation of local turbulence and high-pressure zones. This technique plays a crucial role in improving gelation properties, allowing for precise control over gel structure and properties in soy protein. Hence, ultrasonic technology emerges as a prime option for modifying soy protein, opening up novel possibilities for its application in the food industry, pharmaceuticals and various other domains.

Nowadays, SPI modification still faces several challenges, including ensuring consistency of modification results, reducing cost and equipment complexity, and controllability in large-scale production. In order to overcome these difficulties, it requires a combination of process optimization, technological innovation and sustainability [9]. However, most conventional sonication modifications have been performed on commercially or laboratory-extracted SPI [10]. For example, Hu’s [11] study found that high-intensity sonication of SPI increased its surface hydrophobicity and free sulfhydryl content, and promoted the formation of gel networks. This is consistent with the findings of Zhang et al. [12] who showed that the gel strength and water-holding properties of SPI were significantly improved by 40 min of ultrasound pretreatment, and that the microstructure was more uniform and dense. The effects on functional properties, such as protein gelation, have not been further observed when sonication is performed during the extraction process. Karki et al. [13] first subjected defatted soy flakes to ultrasonic treatment during soy protein extraction, which resulted in enhanced release of proteins and sugars in the extract, leading to increased yields of both components. However, they did not further investigate the impact on protein gel properties. Moreover, ultrasonic modification of SPI after extraction may lead to increased costs. Thus, the hypothesis is proposed that it would be possible to modify SPI by combining ultrasound with the extraction process. To address these issues, a series of experiments was conducted exploring the impact of introducing ultrasound treatment at the alkali solubilization stage of SPI.

This work aimed to study the microstructural and physicochemical impact of ultrasound therapy at varying intensities and durations on the of SPI properties by combining the alkaline solubilization stage of SPI extraction with ultrasonic treatment. Subsequently, the effects of ultrasonication on its TGase-induced gel properties, including structure, strength, water-holding capacity and rheological properties, were evaluated. Finally, the mechanism of the difference between the traditional and current modification methods of SPI by ultrasound was studied. This investigation not only illuminated the refined extraction process but also underscored the benefits of incorporating ultrasound treatment in the alkaline solubilization phase to augment the overall functional properties of SPI.

## 2. Materials and Methods

### 2.1. Materials

Defatted soy flakes were obtained from Shandong Yuwang Ecological Food Industry Co., Ltd., Dezhou, China; the moisture content was 5.0% (*w*/*v*) on wet basis. Soluble protein kit was bought from Nanjing Jiancheng Bioengineering Institute, Nanjing, China; it contains a protein standard solution at a concentration of 0.524 mg/mL. TGase (for SPI, 100 U/g) was purchased from Solarbio Bio-Technology Co., Ltd., Beijing, China. The remaining chemicals were collected as analytical-grade reagents from Sinopharm Chemical Reagent Co., Ltd., Beijing, China.

### 2.2. Preparation of Ultrasonic Modified SPI (UM-SPI) during Extraction Processing

Defatted soy flakes were dissolved in deionized water in 100 mL beaker (10%, *w*/*v*). After adjusting the pH of the solution to 8.0 with NaOH (2 mol/L), it was agitated for 1 h at room temperature; the ultrasound processor with a probe was used to ultrasonic soy flakes dispersion in a 50 mL beaker. The dispersion was placed in the ice water bath during the sonication processes in order to maintain the sample temperature between 10 and 20 °C. The samples were sonicated with an ultrasonic cell pulverizer (Scientz-ⅡD, Scientz, Zhejiang, China) at 200 W, 400 W or 600 W for 60 s, 120 s, 180 s, or 300 s (pulse duration of 1 s for off-time and 5 s for on-time). Next, the supernatant was extracted by centrifuging all samples for 25 min at 6406× *g*, and the pH was adjusted to 4.5. After storage at 4 °C for 30 min, the mixture was centrifuged for 15 min at 1857× *g*. The precipitate was then dissolved in distilled water at a 1:6 ratio, and the pH was brought to 7.0. For later usage, the ultrasonic SPI solution was frozen dry (freezing temperature −70 °C, freeze-drying 80 h) and kept in the fridge.

### 2.3. Preparation of UM-SPI after (UMA-SPI) Protein Extraction

Defatted soy flakes were dissolved in deionized water in a 100 mL beaker (10%, *w*/*v*). After conditioning the solution’s pH to 8.0, it was agitated at room temperature for 1 h. Next, the supernatant was extracted by centrifuging all samples for 25 min at 6406× *g*. The remaining steps are consistent with Section 2.2. The lyophilized SPI was dissolved in deionized water in a 100 mL beaker (10%, *w*/*v*) and performed by ultrasonication at 200 W, 400 W, or 600 W for 180 s (pulse duration of 1 s for off-time and 5 s for on-time) as above procedures, followed by freeze-drying (freezing temperature −70 °C, freeze-drying 80 h) for preservation.

### 2.4. Physicochemical Properties of Ultrasonic-Modified SPI (UM-SPI)

#### 2.4.1. Extraction Rate of Ultrasonic Modified SPI

The soy flake was dissolved in deionized water to prepare the defatted soy flake dispersion (10%, *w*/*v*). The UM-SPI was extracted as described in Section 2.2, and the freeze-dried SPI sample was collected and weighed. Extraction rate (%) was determined by Equation (1):

(1) $$\mathrm{Extraction}\;\mathrm{rate}\;(\%)=\frac{W_{1}}{W_{2}}\times 100\%$$

where *W*_1_ is the weight of the lyophilized SPI, and *W*_2_ is the weight of the soy flake.

#### 2.4.2. Soluble Protein Content

With a little modification, the Sun et al. [14] methodology was used to assess the soluble protein content. An aqueous solution containing 1% *w*/*v* UM-SPI was prepared. After the mixture was agitated for an hour at room temperature, it was frozen centrifuged for 10 min at 10,955× *g*. Next, a spectrophotometer (UV-9000, Metash Instruments Co., Ltd., Shanghai, China) was used to quantify the protein absorbance in the supernatant at 595 nm, and the protein content was ascertained using the Coomassie Brilliant Blue method. An amount of 50 μL of supernatant was taken and mixed with 3 mL of Coomassie Brilliant Blue staining solution in a standard. The mixture was allowed to stand for 10 min before measuring the absorbance at 595 nm. The substitution of the supernatant with the protein standard solution is used as the measurement. Distilled water was used as a blank instead of protein. The SH content of SPI was calculated by following Equation (2):

(2) $$\mathrm{Soluble}\;\mathrm{protein}\;\mathrm{content}\;(\mathrm{mg}/\mathrm{mL})=\frac{A_{m}-A_{b}}{A_{s}-A_{b}}\times C$$

where *A*_m_ represents the absorbance value of measuring tube, *A*_b_ represents the absorbance value of blank tube, *A*_s_ represents the absorbance value of standard tube and C indicates the concentration of the protein standard solution, which is 0.524 mg/mL.

#### 2.4.3. Free Sulfhydryl (SH) Content

The free SH contents of both SPI and UM-SPI were determined using the methodology described in Boatrig et al. [15]. Phosphate buffer (1 × 10^−3^ mol/L EDTA, 1% *w*/*v* SDS, pH 8.0) was used to prepare the SPI sample solution (10%, *w*/*v*). It was centrifuged at 9704× *g* for 10 min. After the supernatant was transferred to the same volume of the previously described phosphate buffer, 0.1 mL of DTNB (0.4%, *w*/*v*) solution was added and mixed rapidly. After that, the mixture was left to settle at 25 °C for 1 h. The mixtures were then centrifuged for 30 min at 9704× *g*, and a spectrophotometer (UV-9000, Metash Instruments Co., Ltd., Shanghai, China) was used to measure the absorbance of the supernatant. The phosphate buffer was used as a control. The SH content of SPI was calculated by the following Equation (3):

(3) $$\mathrm{SH}(\mu \mathrm{mol}/g)=\frac{73.53\times A412\times D}{C}$$

where 73.53 = 10^6^/(1.36 × 10^4^), 1.36 × 10^4^ is the DNTB reagent’s molar absorptivity, *A*412 is the absorbance at 412 nm, D is the dilution ratio and C is the sample’s final concentration.

#### 2.4.4. Surface Hydrophobicity (H_0_)

According to Hu et al. [16], 1-anilino-8-naphthalene-sulfonate (ANS (Sigma Chemical Co., St. Louis, MO, USA)) was employed as a fluorescent probe in the procedure to evaluate surface hydrophobicity. The UM-SPI (0.67%, *w*/*v*) was dissolved in phosphate buffer (pH 7.0). After centrifuging the solutions for 15 min at 9704× *g*, the quantity of soluble protein in the supernatant was measured using the Coomassie brilliant blue technique. The intensity of the fluorescence was measured by fluorescent spectrophotometer (F-2500, Hitachi, Tokyo, Japan). The wavelength was selected at 484 nm for the emission and 365 nm for the excitation, respectively. The relative fluorescence intensity’s initial slope in relation to UM-SPI was determined to calculated the H_0_.

#### 2.4.5. Fluorescence Spectroscopy

Following the method of Maneephan et al. [17], protein tryptophan emission fluorescence spectra were measured using a fluorescence spectrophotometer (F-2500, Hitachi Ltd., Tokyo, Japan). The phosphate buffer (pH 7.0) was added, and the UM-SPI sample was stirred for 2 h. The Bradford (Nanjing Jiancheng Bioengineering Institute, Nanjing, China) reagent kit was used to measure the protein concentration. The excitation and emission slit widths were both 5 nm, and the emission wavelengths were varied from 290 to 450 nm. The excitation wavelength was 280 nm. The buffer was used as control.

#### 2.4.6. SDS-PAGE Analysis

The protein distribution was determined by an electrophoresis apparatus (Mini-Protean Tetra Cell, Bio-Rad, CA, USA). A stacking gel with a 5% (*w*/*v*) concentration and a separating gel with a 12% (*w*/*v*) concentration were utilized in the electrophoresis process. The electrode buffer consisted of glycine (0.192 mol/L), Tris base (0.025 mol/L), and SDS (0.1%, *w*/*v*) at pH 8.3. The sample solution, prepared in Tris-HCl buffer (0.01 mol/L, pH 8.0), contained 2% (*w*/*v*) SDS, 10% (*w*/*v*) glycerol and 0.02% (*w*/*v*) bromophenol blue. Additionally, 5% (*w*/*v*) β-mercaptoethanol was added to the buffer to maintain a reduced state. Electrophoresis was conducted on a 1 mm gel plate with a sample loading volume of 10 μL. Tris-HCl buffer (1 mol/L, pH 8.8) was used to dissolve the material (0.2%, *w*/*v*). It was then immediately injected into an electrophoretic tube after heating for 3 min in a boiling water bath and cooling in an ice bath. The current was adjusted to 20 mA for concentrated gel and 40 mA for separated gel, respectively. Then, gel was stained by Coomassie brilliant blue for 3 h and decolorized by using 10% (*w*/*v*) methanol and 7.5% (*w*/*v*) acetic acid for 10 h.

#### 2.4.7. Fourier Transform Infrared Spectroscopy

Fourier transform infrared spectroscopy was determined with reference to Ma et al. [18]. The UM-SPI samples’ infrared spectra were captured using Fourier transform infrared spectroscopy (TENSOR 27, Buruker, Karlsruhe, Germany) in the 4000–600 cm^−1^ range. At room temperature, a dry environment containing approximately 100 mg of SPI sample was combined with KBr, and its spectrum was collected at a resolution of 4 cm^−1^ with co-addition of 64 scans. Potassium bromide thin sheets without samples were used as blank. All spectra were baseline-corrected, and their protein amide I region was analyzed by Fourier self-deconvolution (FSD) and peak separation by OMNIC32 and PeakFit V4.12 software. The secondary structures of the SPI were calculated by the peak area assigned to its structures of the FTIR spectrum.

### 2.5. Preparation of TGase-Catalyzed Soy Protein Isolate Gel (TCSG)

A solution of modified soy protein with a concentration of 10% (*w*/*v*) was prepared using distilled water, and then stirred magnetically for 2 h. After adding 40 μ/g of TGase enzyme to the mixture, it was incubated in a water bath at 45 °C for 2 h before being inactivated at 90 °C for 10 min. The gel was then promptly cooled to room temperature in an ice bath and stored at 4 °C for future use.

### 2.6. Gel Properties of UM-SPI or UMA-SPI

#### 2.6.1. Gel Strength Analysis

Gel strength of TCSG was measured according to Hu et al. [11]. At room temperature, the gel was first balanced for 30 min. The strength of the gel was then measured using a texture analyzer (TA. HD plus, Stable Micro System, London, UK) equipped with a P/0.5 probe type. The probe was set to move at a speed of one millimeter per second with a trigger force of five grams. The data collection rate was 200 PPS. The gel strength is expressed in terms of hardness, i.e., the maximum induced force (g) during the downward pressure of the probe.

#### 2.6.2. Water Holding Capacity (WHC)

The WHC was assessed using a slightly altered version of the Zhu et al. [19] methodology. Samples (3 g) were placed in the 50 mL centrifuged tubes and centrifuged at 7430× *g* for 20 min at 4 °C to remove the water. WHC was calculated as the following Equation (4):

(4) $$\mathrm{WHC}\;(\%)=\frac{W_{t}-W_{r}}{W_{t}}\;\times 100\%$$

where *W*_r_ is the weight of water that spills out of the gel after centrifugation (g) and *W*_t_ is the total weight of water in the gel sample (g).

#### 2.6.3. Rheological Measurements

Rheological measurements were carried out according to the method of Zhang et al. [20] with some modifications. The apparent viscosity and dynamic viscoelasticity of the gel were measured by a rheometer (Physica MCR 301, Anton Paar, Graz, Austria) based on the method of Sittikijyothin et al. [21] with some modifications. SPI solution (10.0%, *w*/*v*) and TGase (40 u/g) were mixed together on a parallel plate with dimensions of 40 mm, a gap of 1 mm, and a strain of 0.05%. To induce gel formation, the material was heated to 45 °C for 2 h, then cooled to 25 °C. Parameters reflecting changing storage modulus (G′) and loss modulus (G″) were recorded during the formation of the sample.

#### 2.6.4. Scanning Electron Microscopy (SEM)

Rheological measurements were carried out according to the method of Zhang et al. [12]. After being frozen for two days, each sample was sliced into 0.1 cm-diameter squares. Before being examined under a SEM, samples were coated in gold or palladium and critical-point-dried. Using a 10 kV voltage, the protein gel’s microstructure was seen using a scanning electron microscope (SU8010, Hitachi, Tokyo, Japan) [22].

### 2.7. Statistical Analysis

With the exception of what is stated, each measurement was made in triplicate. An analysis of variance (ANOVA) and Duncan’s test were used to determine the statistical significance of differences (*p* < 0.05) among means using SPSS version 27.0.

## 3. Results and Discussion

### 3.1. Extraction Rate

The effect of ultrasound treatment on the extraction rate of SPI during the extraction process is shown in Table 1. The extraction rates of modified SPI were notably improved by the ultrasound treatment in comparison with that of control, and it significantly increased from 24.68% to 42.25% with the increase in ultrasonic power and treated duration. This trend was consistent with Karki’s [13] research, who discovered that defatted soy flakes’ particle size was significantly reduced by ultrasonic processing. It may be attributed that the cell walls of the soy flakes were disrupted by ultrasound, and the release of intracellular substances in flakes were released in leached solution, leading to significantly increasing of protein production. In addition, the alkali solubilization stage could allow the tight structure of soy meal to become loose. Meanwhile, the alkali condition induced a destructive effect on the secondary bonds of protein molecules, especially the hydrogen bond, in which certain polar groups were dissociated and the same charge of the molecule surface was attached, resulting in promoting the separation of conjugates and proteins and solubility of the protein. At the same time, the mechanical vibrations induced by ultrasound increased the contact area between the sample and the alkaline solution. It assisted the solubilizing effect of alkali extraction on protein molecules in increasing extraction rate [23]. Analogously, Yang et al. [24] reported the increased extraction rate of rice protein by ultrasonic treatment. They found that ultrasound combined with α-amylase degradation treatment significantly increased the extraction of rice protein. This is because ultrasonography has two ways in which it might improve mass transfer; some starch-protein structures can be broken apart by it. On the other hand, it can enhance the interaction between amyloid-protein agglomerates and α-amylase degradation, thereby amplifying starch degradation. This is similar to the way in which ultrasound combined with alkali treatment in this experiment improved the extraction rate of soy protein. Hence, combining ultrasound processing as a green and reliable method could be combined into SPI extraction processing to effectively raise extraction rate and reduce the chemical reagents amount [25,26,27].

### 3.2. Soluble Protein

**Figure 1:**
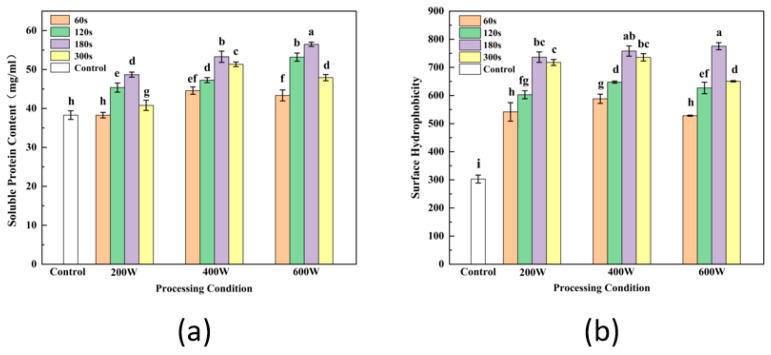
The effect of ultrasound treatment on soluble protein content (**a**) and surface hydrophobicity (**b**). Letters above each bar indicate significant differences between treatment conditions.

Soy protein solubility reflects the proportion of soluble proteins in SPI as peptide bonds or side chain groups interact with water molecules [28]. The impact of ultrasound treatment on soluble protein content is illustrated in Figure 1a, revealing a substantial increase in soluble protein levels (*p* < 0.05) compared to the control. It exhibited a pattern of initial growth followed by a subsequent decline as the treatment duration extended. This is consistent with the study of Lee et al. [29], who found protein solubility of SPI treated with alkali followed by ultrasound treatment for 5 min was significantly increased from 1.49% to 82.73%, which was attributed to the changed conformation of some proteins inducing more insoluble protein to become soluble during ultrasonic treatment. A similar effect on solubility of milk protein was obtained when sonication was performed under alkaline solubilization conditions. This was because proteins under extremely alkaline circumstances could be subjected to sonication, which exposed several polar groups and further expanded the protein chain, resulting in good solubility [23,30]. On the other hand, increasing the solubility of SPI by sonication might be explained in terms of decomposing the large protein aggregates into smaller protein molecules and making proteins more easily dispersed in water, where the reduction in sample particle size favors protein–water interaction [10]. Thus, the ultrasound-assisted treatment could be demonstrated to effectively improve protein solubility and transform protein structure.

### 3.3. Free SH Content

Free SH content is one of the important factors affecting protein functional properties, such as stability, antioxidant properties, etc. [11]. Table 2 shows the significant increasing of free SH content as ultrasound pretreated in extraction processing (*p* < 0.05). The content of free SH in natural SPI is 4.00 μmol/L. Free SH content of SPI effectively rose with the increased ultrasound intensity and time extended, reaching a maximum value of 6.83 μmol/L at an intensity of 600 W for 300 s (*p* < 0.05). This phenomenon could be attributed to the disruption of intermolecular disulfide bonds by ultrasound. A previous study by Hu et al. [3] similarly found that the free SH group content of SPI rose with increasing ultrasound intensity and time. A plausible explanation for these findings could be that the cavitation event that occurs during the ultrasonic process contributes to the creation of SH groups and the decrease in disulfide bonds. Simultaneously, the SPI is denatured and the SH residues embedded in the nonpolar region of the SPI are exposed to the molecular surface due to the large shear force and pressure generated by ultrasound [31]. Consistent results could also be found in the study of modifying egg white protein using a pulsed electric field [32]. Another explanation is that the distribution of cysteines within the protein molecule is determined by the specific structure and conformation of the protein [33]. Generally, cysteines interact with other amino acid residues to form disulfide bonds, thereby stabilizing the tertiary and even quaternary structure of the protein [34]. Ultrasonication, a commonly employed physical modification technique, can cause protein denaturation by generating high-frequency vibrational waves that result in the displacement and collision of protein molecules [3]. This process often leads to alterations in the secondary and tertiary structures of SPI, and sometimes even affects its quaternary structure, thereby exposing more free sulfhydryl groups and promoting hydrophobic interactions [35]. In addition, the effects the oxidation–reduction state of cysteine by ultrasonication need to be taken into consideration, which contribute to being more prone to forming free SH groups in SPI.

The results demonstrate that the mechanical vibration and eddy current effects of sonication led to changes in the overall folding state of SPI, exposing more internal hydrophobic structures, resulting in an increase in SH groups on the molecular surface.

### 3.4. Surface Hydrophobicity (H_0_)

Surface hydrophobicity (H_0_) is associated with the hydrophobic groups on the protein surface and the conformation of proteins formed through non-covalent intermolecular interactions. This influences the functionality, stability and gel properties of SPI [36]. As shown in Figure 1b, the H_0_ of SPI effective improved after ultrasonic treatment. A significant raising of H_0_ was observed with the increased ultrasonic time (*p* < 0.05), which indicates that ultrasound resulted in an increased exposure of hydrophobic residues on the protein surface, causing further unfolding of the protein conformation and greater extension of the protein molecule. Jiang et al. [37] found that higher pH during the alkaline solubilization phase also led to an increased electrostatic repulsion within the SPI molecule and protein resolution, thus exposing hydrophobic amino acid residues. These findings were in agreement with the increasing of the free SH content above. There is a significant linear positive correlation between H_0_ and free SH content, reflecting that the exposure of sulfhydryl groups inside of the inner protein structure is one of the explanations for increased H_0_ [38,39]. Oliveira et al. [40] also reported that the H_0_ of the sonicated pea protein was significantly enhanced. Many hydrophobic groups embedded inside protein molecules or polymers were highly efficiently exposed by the hole effect and micro beam effect of ultrasound. However, longer time of ultrasound could reduce the H_0_ due to the protein re-aggregating and re-embedding of hydrophobic groups [18].

### 3.5. Fluorescence Spectroscopy

**Figure 2:**
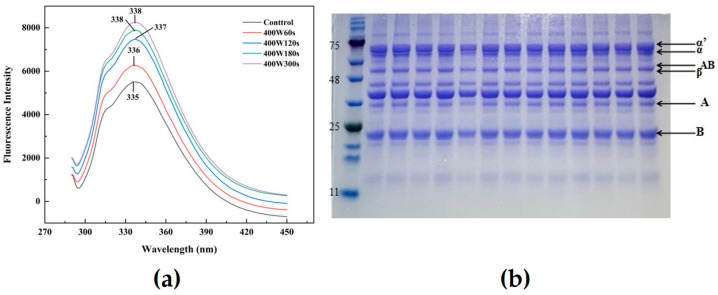
Changes in SPI fluorescence spectra after 400 W ultrasonic treatment as an example (**a**); gel electrophoresis (**b**) (from left to right, marker, native SPI, 200 W, 400 W and 600 W are processed for 60, 120, 180 and 300 s, respectively. The number labelled next to the leftmost band refers to the molecular weight size of the marker. The subunits of SPI are labelled next to the rightmost band).

Endogenous fluorescence spectra of proteins are obtained by determining the polarity of the environment in which the tryptophan residues are located. It can reflect changing of protein conformation and interactions between proteins [41]. Figure 2a showed that the fluorescence intensity of ultrasound-modified SPI was significantly enhanced compared to the control. Their fluorescence intensities exhibited notable increase as well as the λ_max_ values with extending the ultrasonic time (*p* < 0.05). It implied that parts of the protein structure appeared to unfold in exposing more Trp (tryptophan)/Tyr (tyrosine) residues as well as hydrophobic groups by the cavitation effect of ultrasound, thereby increasing the non-polarity of protein conformation in the microenvironment, corresponding to the results of surface hydrophobicity. Hu et al. [42] verified that when the polarity of the solvent environment in which the protein tryptophan residues are located increases, the tryptophan residues were induced to shift from the internal hydrophobic region of globular proteins to the surface exposed to the solvent environment, leading to the increased λ_max_. He et al. [43] also reported the maximum emission wavelength of SPI increased by 2 nm after sonication, which demonstrated how the application of ultrasonic pretreatment stretched the tertiary structure of proteins and interfered with their ability to interact. Therefore, ultrasonication during the extraction process could increase the exposure of inner residues within soy protein and trigger more stretching of protein molecules, resulting in transforming the tertiary and quaternary structure of SPI.

### 3.6. Fourier Transform Infrared Spectroscopy

**Figure 3:**
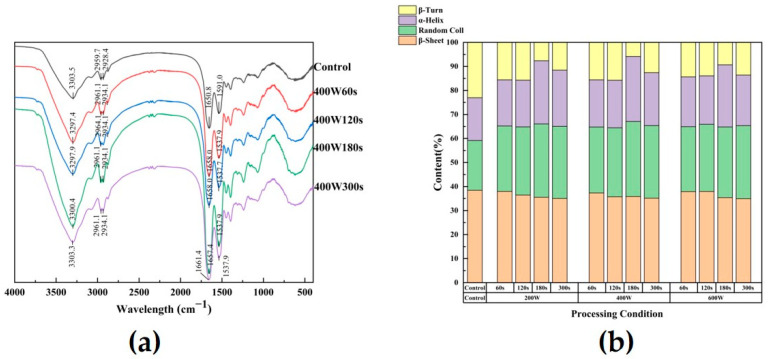
Changes in SPI infrared spectra after 400 W ultrasonic treatment as an example (**a**); influence of different ultrasonic intensity and time on secondary structure of SPI (**b**).

Fourier transform infrared spectroscopy was employed to investigate the modifications induced by ultrasonic therapy in the secondary structure of SPI, along with the characterization of functional groups. Their ATR-FTIR spectra are displayed in Figure 3, together with the control. The -OH stretching vibration is responsible for the absorption peak located at 3303.5 cm^−1^, while the absorbance in the range of 2800–3000 cm^−1^ corresponds to the stretching vibrations of C-H bonds; the peak formed at 2928.4 cm^−1^ is related to the quantity of free SH in our study [44]. With the increase in ultrasonic treatment time, the wavenumber of the peak redshifted from 2928.4 cm^−1^ to 2934.1 cm^−1^. It may be attributed to the location of aliphatic amino acids on the protein surface and more interaction (i.e., electrostatic interaction) appeared, which changed the hydrophobic interactions in the asymmetric stretching vibration of –CH_3_ and -SH. The rise in these peak intensities by ultrasound also indicated the increased content of free SH, consistent with the results in Section 3.3. The absorption peaks at these two locations were little impacted by the ultrasonic treatment. When examining the structural alterations in proteins, the peak position differences of the amides I region from 1700 to 1600 cm^−1^, II region from 1530 to 1550 cm^−1^, and III region from 1260 to 1300 cm^−1^ were typically utilized [45]. Taking 400 W ultrasound as an example (Figure 3a), the absorption peaks were shifted from 1650.8 cm^−1^ and 1591.0 cm^−1^ (control variables) to 1658.0 cm^−1^ and 1537.9 cm^−1^ (60 s), 1658.0 cm^−1^ and 1537.7 cm^−1^ (120 s), 1657.4 cm^−1^ and 1537.9 cm^−1^ (180 s), and 1661.4 cm^−1^ and 1537.9 cm^−1^ (300 s). This suggested that the protein’s secondary structure was altered to variable degrees by ultrasonic treatment at various frequencies. To gain deeper insights into the protein secondary structure alterations in these modified SPI samples, an in-depth analysis of the amide I band was carried out through Fourier self-deconvolution (FSD). The peak areas associated with each type of secondary structure was computed and displayed in Figure 3b. In contrast to the secondary structure of untreated SPI, the ultrasound-treated SPI exhibited a significant increase in random coil and α-helix, accompanied by a reduction in β-sheet and β-turn as the treatment duration extended. This is in agreement with the findings of Huang et al. [46], which showed that the content of α-helix gradually increased and a gradual decrease in β-sheet with decreasing ultrasound time. This phenomenon occurs due to the modification of the protein’s conformation induced by sonication, leading to a decrease in β-sheet content and the formation of a random coil structure (*p* < 0.05). The decrease in β-sheet content may be due to external forces such as mechanical shear force, temperature and pressure that cause the protein structure to partially unfold. Ultrasonic treatment disrupted the hydrogen bond, unfolded molecules, and subsequently formed a random coil structure [47]. In conjunction with the findings in Section 3.1, alkaline environments have a disruptive effect on the secondary bonds of proteins. Consequently, the application of ultrasonication during the extraction of SPIs in alkaline environments induces partial unfolding of β-sheet and β-turn structures, transforming them into α-helix and random coil structures in response to applied forces [48].

### 3.7. Polyacrylamide Gel Electrophoresis

SDS-PAGE can effectively reflect the differences in SPI subunit composition between modified SPIs and the control group. The reducing chemical mercaptoethanol has the ability to break disulfide bonds and hydrophobic interactions between SPI subunits, which might result in subunit dissociation. According to the polyacrylamide gel electrophoresis diagram (Figure 2b), SPI showed typical 11S globulin and 7S globulin. There was no significant difference in the electrophoretogram of soybean globulin subunits after ultrasound treatment, indicating the non-effectiveness of sonication in altering individual subunits structure of the protein. This was consistent with findings of some researchers [3,18], whose results showed that there were no significant changes in various subunit groups of SPI through the ultrasound treatment.

### 3.8. Gel Strength Analysis

**Figure 4:**
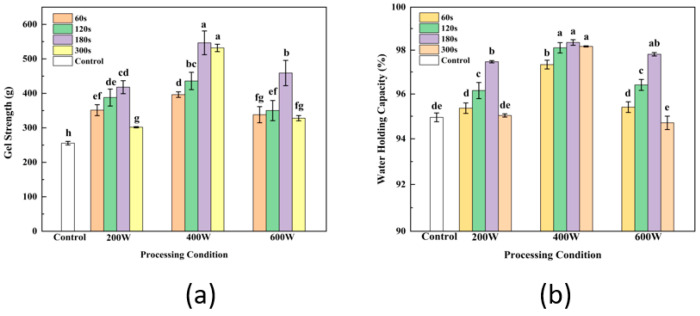
Effect of different ultrasonic intensity and time on SPI gel strength (**a**) and water holding capacity (**b**). Letters above each bar indicate significant differences between treatment conditions.

One of the most crucial characteristics of gel is its strength, which is a reflection of its superior network structure. The effects of ultrasonic power and time on strength of gel are shown in Figure 4a, respectively. The results demonstrate the obvious improvement of gel strength by ultrasonic treatments in comparison with SPI gel without ultrasonic treatment during the extraction. As the treated time and power of ultrasound increased, the strength of TGase-induced SPI gel showed a significant increase at first and decrease later (*p* < 0.05). Ultrasound of soy solution at 400 W for 180 s during SPI extraction resulted in a significantly higher strength value of SPI gel (*p* < 0.05). Zhang and Hu et al. found that ultrasonication significantly increased the gel strength of TGase and CaSO_4_-induced SPI gels [11,12]. This phenomenon might be due to increased protein solubility and free sulfhydryl content by ultrasound therapy, which is consistent with the free sulfhydryl concentration trend found in our earlier findings [49]. Free SH groups could oxidize to form disulfide bonds and promote the formation of aggregates, resulting in an enhancement of the gel texture during gel formed. The study of Zhang et al. [20] found that one reason for the enhanced strength of SPI gels as a result of ultrasonication is that ultrasonic pretreatment increased protein solubility and exposed more enzyme-catalyzed sites, leading to a more homogeneous and dense gel network in US-TCSG. Meanwhile, disruption of the secondary structures of soy protein by pretreated ultrasound could lead to unfolding of SPI molecules and more action sites for TGase enzymes, and consequently, more enzymatic cross-linking modification of soy protein gel favoring increasing gel strength of pretreated SPI [50]. However, excessive ultrasound can reduce the strength of gel. Excessive ultrasound causes partial aggregation of proteins, increasing cross-linking reactions between sulfhydryl groups, which further leads to loss of sulfhydryl groups or formation of non-reducing disulfide bonds. Meanwhile, a decrease in the content of free SH groups and formation of fewer disulfide bonds happened, contributing to inhibit the catalytic crosslinking of enzymes and the suppressed formation of ε-(γ-glutamyl) isopeptide bonds of glutamate lysine.

### 3.9. WHC Analysis

The effect of ultrasound treatment of WHC of gel also followed the same trends as observed in gel strength (Figure 4b). The WHC of gel pretreated by ultrasound at 400 W were dramatically higher than that of gel treated with other ultrasound power (*p* < 0.05), but were not statistically different among treatment times (*p* > 0.05). The dense and uniform spatial structure of gel is conducive to the binding of gel to water molecules. Similar results were found by Zhang et al. [12], who discovered that SPI caused by ultrasound created a denser, more uniform, more stable gel network. SPI molecules’ intrinsic active groups were exposed, and their conformation was altered by ultrasound. The influence of ultrasound on the water-holding capacity (WHC) and strength of SPI gel could promote the development of a more uniform, dense and finer-pored gel network through enzyme-catalyzed gelation. Consistently, excessive sonication might contribute to the formation of aggregates of proteins that were not uniformly distributed in the gel structure as well as loose structure, resulting in voids within the gel and difficulty to retain water [51].

### 3.10. Rheological Measurements

**Figure 5:**
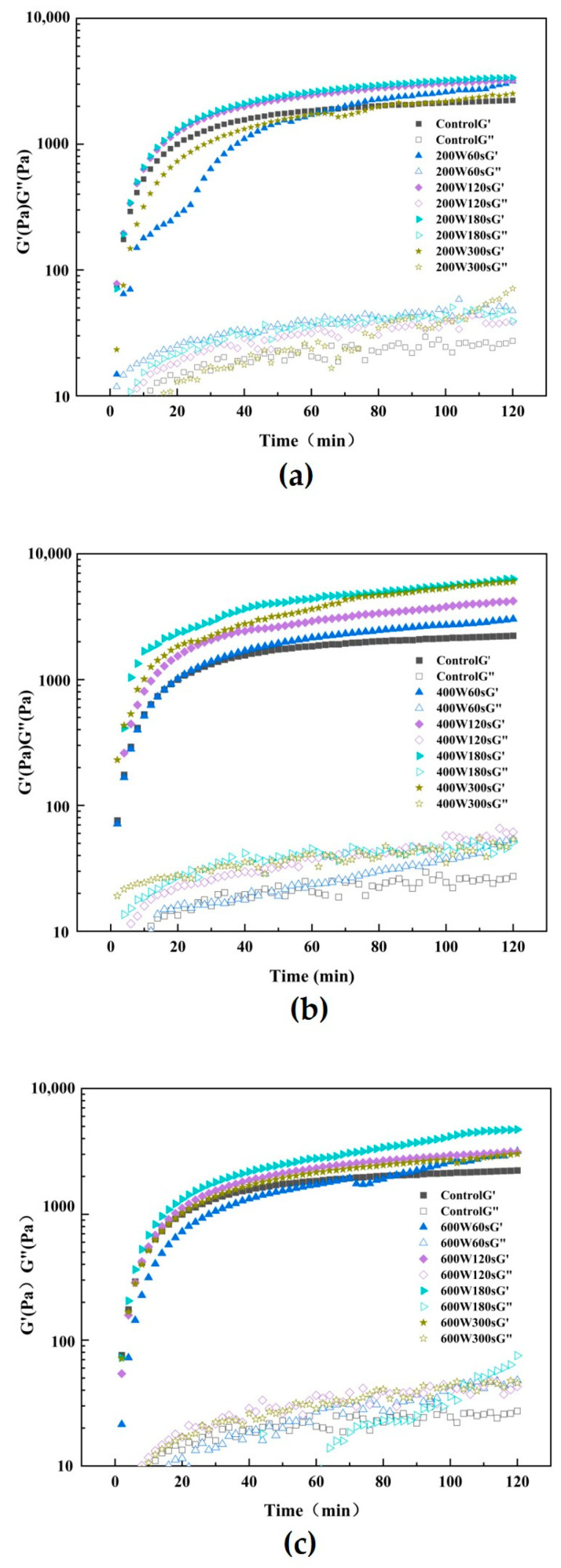

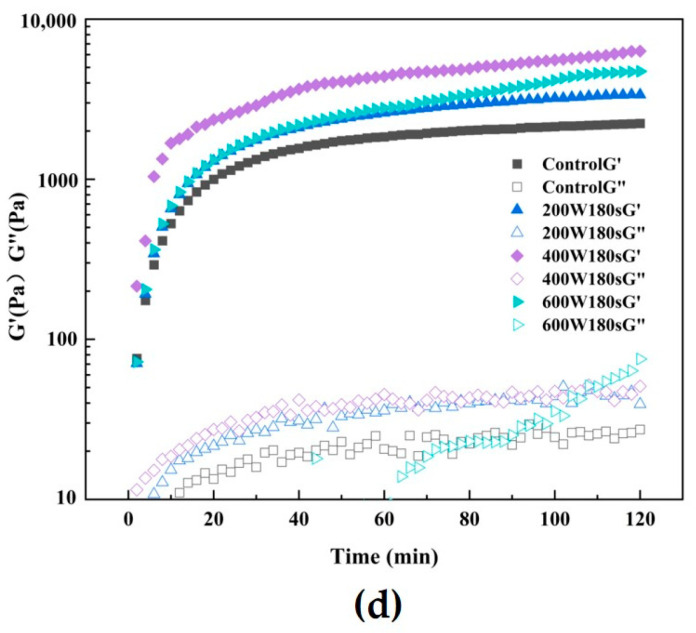
Trend of untreated and 200 W (**a**), 400 W (**b**) and 600 W (**c**) ultrasonic treatment and three power ultrasound 180 s G ‘/G’ (**d**) over time.

Using a rheometer with a temperature sweep pattern, the impact of various ultrasonic treatments during extraction on the gelation network of TGase-induced SPI gel was examined. Figure 5 depicts the progression of the gel’s storage modulus (G’) and loss modulus (G”). The viscoelasticity of SPI gel catalyzed by TGase enzyme was found to be improved by ultrasonic pretreatment, as evidenced by the considerably higher G’ and G” values of the ultrasound-pretreated gel compared to the control group (*p* < 0.05). The G’ values of all gel samples were greater than the G” values, indicating that all gel samples exhibited elastic solid properties [52]. In addition, the G’ value increased with increasing ultrasound time at the same ultrasound intensity [53]. Similarity, Tang et al. [53] found the G’ value of SPI treated with ultrasound is much higher than that of G”, and the untreated SPI dispersion induced by TGase formed a more viscous gel, while the ultrasound induced the SPI dispersion to form into a more elastic gel. It implied that ultrasonic pretreatment improved the viscoelasticity of TGase SPI gel. Soluble aggregates, which are typically thought of as intermediates of heat-induced SPI gel formation, could represent the mechanism underlying the enhanced effect of ultrasonic on gelation. Furthermore, Figure 5 (d) shows the rheological property of gel made by ultrasonic-pretreated SPI at 200 W, 400 W, and 600 W for 180 s, respectively. The results show the gel treated by ultrasound at 400 W for 180 s exhibited the highest elastic modulus in comparison with other treatments, which demonstrates that ultrasound can effectively promote the viscoelasticity of gel under this condition. On the one hand, ultrasound induces structural changes within protein molecules, resulting in more tightly arranged molecules. This tightening effect may lead to enhanced interactions between the protein molecules, thus increasing the overall modulus of elasticity. On the other hand, in combination with our results of FTIR above, sonication leads to changes in the secondary structure of proteins, such as rearrangement of α-helices or β-sheets [54]. Such structural changes might have an impact on the mechanical properties of the protein, including an increase in the elastic modulus. The comprehensive promotive effect of structural changes by ultrasound led to exhibition of higher elastic modulus of proteins, resulting in a better elasticity and stability. Zhao et al. [55] suggested a more uniform network and higher density of TGase-catalyzed gel associated with ultrasonic treatment.

### 3.11. Scanning Electron Microscopy (SEM)

**Figure 6:**
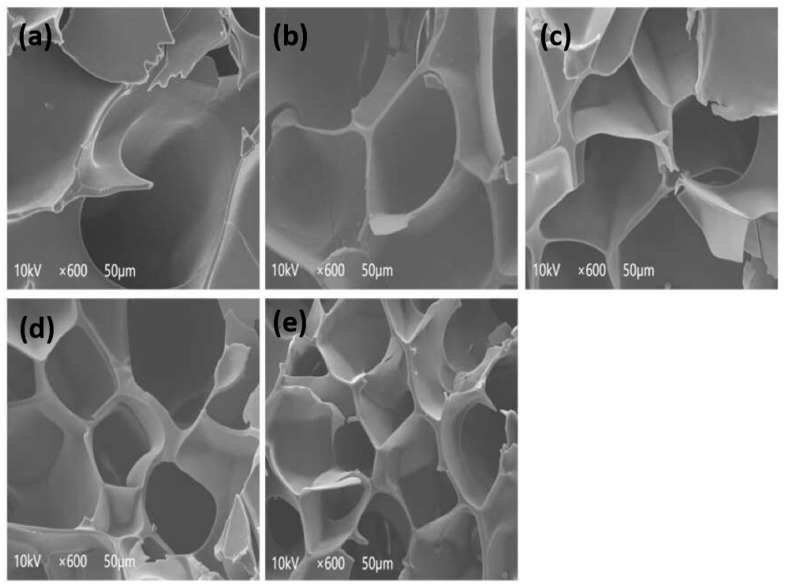
Microstructure of SPI gel (control (**a**), 400 W ultrasound 60 s (**b**), 400 W ultrasound 120 s (**c**), 400 W ultrasound 180 s (**d**) and 400 W ultrasound 300 s (**e**), from top to bottom).

The impact of ultrasonic waves on the microstructure of SPI gel during the SPI extraction process was examined using scanning electron microscopy. Figure 6 shows the network structures of SPI gel induced by TGase after ultrasonic treatment with different power and times. Larger pores, low cross-linking degree and unevenness were observed in the network structure of gel without ultrasound. Conversely, denser and more uniform network structures with smaller pores appeared as ultrasound treatment time increased. Gels made from SPI pretreated with different ultrasonic powers exhibited the similar trend. A tighter and more uniform gel network may emerge as a result of greater intermolecular S-S bonding and hydrophobic interactions encouraged by the higher SH and hydrophobic group content on the surface of the SPI molecules above. These results indicate that ultrasound played a pivotal role in significantly improving the homogeneity of the microstructure in SPI gel, resulting in decreased particle size and heightened molecular interactions. Furthermore, ultrasonication created additional bonding sites, fostering the formation of intermolecular ε-(γ-glutamyl) lysine cross-links and enhancing the TGase enzyme cross-linking extent. This led to an increased number of polymer aggregates formed during the gelation process [12]. The gel formed by ultrasonically treated SPI exhibited a more compact and uniform gel network with consistent pores and a filamentous structure, aligning with the observations made by Hu et al. [11].

### 3.12. Comparison of Gel Properties Modified by Ultrasound before and after SPI Extraction

**Figure 7:**
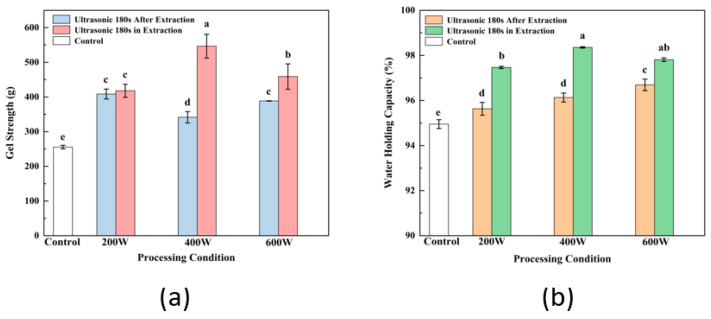
Gel strength (**a**) and WHC (**b**) of ultrasonically modified gels before and after SPI extraction. Letters above each bar indicate significant differences between treatment conditions.

After extraction, ultrasonic treatment of SPI is typically used as a modification technique to enhance the functional properties of SPI and its gel, such as the solubility, SH content, secondary structure and gel’s ability to hold water. Comparison of gel properties modified by ultrasound before and after SPI extraction are analyzed and shown in Figure 7. According to the comparative analysis of gel strength (Figure 7a) and WHC (Figure 7b) of ultrasound-assisted SPI at different intensity, the gel strength and WHC of the protein prepared by ultrasonic treatment during extraction were significantly higher than those of the control group (*p* < 0.05). Meanwhile, at the same ultrasonic intensity and time, the gel properties of groups treated during the SPI extraction were also dramatically improved compared to groups treated with ultrasound after extraction, which indicated the greater effectiveness of ultrasound during SPI extraction in modifying the structure and increasing the strength and water holding of gel. Karki et al. [13] reported that the proteins were in an extended state, and the expanded protein structure was more susceptible to the influence of ultrasonic treatment during the alkaline dissolution stage of the extraction process. Li et al. [56] applied ultrasound–alkali-assisted pretreatment on rice protein; the results showed the synergistic damage of sonication and alkali resulted in a decrease in residues of hydrophobic groups and an increase in soluble small molecules. The effect of the combination of sonication and alkali denatured the protein structure, which aimed to increases the unfolding of the protein. As the soybean meal compact structure became loose under the alkali condition, the alkali has a destructive effect on the secondary bonds of the protein molecule, especially the hydrogen bond. The mechanism of enhancement of SPI gelation properties was associated with several factors of changes in SPI characteristics, including the dissociation of the polar groups, the same charge on the surface of the protein molecules and the transformation of the SPI secondary structure, which is consistent with the increase in soluble protein content and free SH groups and changes in H_0_. Therefore, the same effectiveness of ultrasonic modification could be reached with less ultrasound intensity and shorter treatment time during the SPI extraction step, leading to lower energy consumption and cost savings.

## 4. Conclusions

In this study, the combination of sonication with the alkaline dissolution stage of SPI extraction effectively improved its extraction rate and SPI characteristics, including the increase in content of soluble proteins, free SH groups, and changes in the H_0_, secondary structure and fluorescence intensity of SPI. The increase in sonication power could expose more hydrophobic residues within the protein core to the molecular surface and transform the microstructure of SPI with breaking of hydrogen bonding and intramolecular interactions, thus providing more binding sites for TGase. Meanwhile, gels prepared using TGase-induced ultrasonic extraction of SPI exhibited significant enhancements in mechanical properties, with SPI extracted at 400 W ultrasound intensity for 180 s demonstrating optimal values for texture, WHC and rheological properties of the prepared gels. Compared with the traditional modification methods of SPI followed by ultrasonication, the combination of ultrasonication during the extraction process was more effective in uniforming the gel structure and strengthening the mechanical properties of gel. In conclusion, the application of ultrasonication at the alkaline dissolution stage of SPI extraction can effectively improve the mechanical properties of TGase-induced SPI gels, and sonication at 400 W intensity for 180 s was found to be the optimal parameter for good SPI gel properties in conjunction with the results of this study. It has the potential for utility as a green technology for SPI extraction and modification that may be an alternative production method of lower energy consumption and cost savings for food manufacturing.

## Figures and Tables

**Table 1:** SPI extraction rates (%) for different processing intensities and times ^1^.

Ultrasound Intensity and Time	60 s	120 s	180 s	300 s
Un-sonicated native SPI	24.68 ^g^ ± 0.40			
200 W	29.35 ^f^ ± 0.31	29.48 ^f^ ± 0.10	31.12 ^e^ ± 0.12	31.25 ^e^ ± 0.00
400 W	33.08 ^d^ ± 0.51	34.60 ^c^ ± 0.03	35.44 ^b^ ± 0.06	35.65 ^b^ ± 0.07
600 W	33.12 ^d^ ± 0.12	35.31 ^b^ ± 0.02	41.62 ^a^ ± 0.26	42.25 ^a^ ± 0.04

^1^ The results in the table are expressed as mean value ± standard deviation, and superscripts in the upper right corner represent significant differences (only those reported by the authors are shown; n ≥ 3).

**Table 2:** Free SH content (μmol/L) of modified SPI by ultrasound during the extraction ^1^.

Ultrasound Intensity and Time	60 s	120 s	180 s	300 s
Un-sonicated native SPI	4.00 ^h^ ± 0.01			
200 W	4.03 ^h^ ± 0.03	4.05 ^h^ ± 0.06	5.13 ^g^ ± 0.00	5.47 ^e^ ± 0.01
400 W	5.31 ^f^ ± 0.01	5.33 ^f^ ± 0.01	5.82 ^d^ ± 0.01	5.42 ^e^ ± 0.01
600 W	5.84 ^d^ ± 0.00	6.34 ^c^ ± 0.03	6.49 ^b^ ± 0.00	6.83 ^a^ ± 0.01

^1^ The results in the table are expressed as mean value ± standard deviation, and superscripts in the upper right corner represent significant differences (only those reported by the authors are shown; n ≥ 3).

## Data Availability

The original contributions presented in the study are included in the article, further inquiries can be directed to the corresponding author.
